# Supplementary material for: Adsorption of oxytetracycline on kaolinite
Source: PLoS One. 2019 Nov 15;14(11):e0225335. doi: 10.1371/journal.pone.0225335 (PMC6857953; doi:10.1371/journal.pone.0225335)
Supplement: Attachment — Submitted filename: Response to Reviewers.doc [file pone.0225335.s006.doc]

Dear editors and reviewers:

We thank you very much for giving us an opportunity to revise our manuscript. We appreciate editors and reviewers very much for their valuable and very helpful comments and suggestions on our manuscript “Adsorption of oxytetracycline on kaolinite” (PONE-D-19-21989). We have studied the comments carefully and tried our best to improve the manuscript and made changes according to the editor’s and reviewers’ comments. The main corrections in the paper and the responses to the editor’s and reviewers’ comments are appended as followed.

**Responds to the editor’s and reviewer’s comments:**

**Responds to the editor**

1. Please ensure that your manuscript meets PLOS ONE's style requirements, including those for file naming. The PLOS ONE style templates can be found athttp://www.journals.plos.org/plosone/s/file?id=wjVg/PLOSOne_formatting_sample_main_body.pdf and <http://www.journals.plos.org/plosone/s/file?id=ba62/PLOSOne_formatting_sample_title_authors_affiliations.pdf>

**Response**: We have modified the manuscript according to PLOS ONE’s style requirements.

2. We suggest you thoroughly copyedit your manuscript for language usage, spelling, and grammar. If you do not know anyone who can help you do this, you may wish to consider employing a professional scientific editing service.

**Response**: Considering the editor’s comment, the manuscript has been edited by AJE.

3. We noticed you have some minor occurrence of overlapping text with the following previous publication(s), which needs to be addressed: * Sorption of tetracycline on biochar derived from rice straw and swine manure, doi: 10.1039/C8RA01454J

**Response**: Some sentences have been modified to avoid overlapping text in the manuscript.

4. PLOS authors have the option to publish the peer review history of their article ([what does this mean?](https://journals.plos.org/plosone/s/editorial-and-peer-review-process#loc-peer-review-history)). If published, this will include your full peer review and any attached files.

**Response**: No

**Responds to the reviewer #1**

1. On Section 2.3 Data analysis: The author mentioned that: "The variation of solution pH was from 3.5 to 11.5, and the concentration of OTC was 5mg/L or 10mg/L. ". Since fine kaolinite will change the pH of the solution, it is recommended to specify whether it is the initial or equilibrium pH of the solution.
 **Response**: The solution pH is the initial pH and we have added the description of pH in the manuscript.

2. On Section 3.4 The effect of pH on adsorption: Kaolinite is a 1:1 layer mineral, in which one layer consists of an alumina octahedral sheet and another consists of silica tetrahedral sheet. The charge characteristics of these two layers and edges vary greatly in solutions with different pH values. Therefore, it is suggested to describe in detail the charged characteristics of kaolinite particles at different pH values. It is helpful for the authors to analyze the effect of pH on adsorption capacity for OTC sorption on kaolinite surface.

**Response**: As the reviewer’s comment, we added the charged characteristics of kaolinite at different pH values inferred from previous studies in the part of discussion. At same time, we discussed how charged characteristics of kaolinite influence the OTC sorption at different pH values.

**Responds to the reviewer #2**

1. The English and language and grammar are poor throughout. This could be improved, this is not a huge job, but it does need to be corrected to improve the clarity and intent of some of the arguments and reduce ambiguity.

**Response**: The English language and grammar has been improved. Discussion part has also been tried to clear.

2. Page 8, why SA to 4 decimal places? The SA is very low compared to many kaolinite samples…why? Large average particle size? Very low porosity? How do we know all of the sand and other contaminating minerals were removed? Is there XRD information?

**Response**: The equipment of measuring SA gave a 4 decimal value. Considering to small value of SA, the SA is kept 2 decimal places. The kaolinite used in this experiment was bought from Macklin (Shanghai) and pretreatment of kaolinite was relatively simple (just filtered through a 300 mesh sieve), so low SA is obtained. We have found that kaolinite SA of 6.45m^2^/g was reported by Yandan Li. Most particle size focused on 2.1um and only porosity of kaolinite of 0.0047cm^3^/g. There is no XRD information for kaolinite sample because of limit of experimental condition.

3. Page 8, what was the pH of the kinetic and isotherm experiments…it would be useful if this was stated in the methods section.

**Response**: The pH of the kinetic and isotherm experiments is 5.5 and manuscript has been revised for this question.

4. Page 8, is there any replication of the experiments? What is the uncertainty, what controls of data quality were used?

**Response**: We have added the contents in section 2.2 in the manuscript.

5. Page 8, what was the ionic strength? The introduction suggests that ionic strength has been tested by other researchers, but there is no mention of the ionic strength for the suspensions in this study.

**Response**: In this study, 0.01 M CaCl_2_ was used as background [electrolyte](javascript:;) of solution and in the manuscript we have added the description of solution background electrolyte.

6. Page 8, How was the pH controlled for the sorption experiments…adjustments were made between pH 3.5 and 11, but was this measured at the beginning or at the end of the equilibration period? Was there any change in the pH over the equilibration time (it would be unusual if the pH did not change a little)?

**Response**: pH was measured at the beginning of the equilibration experiment. The pH changed by 1 to 2 units of pH after equilibration.

7. Page 8, Why would an intra-particle diffusion model be appropriate here, the SA is very low indicating little porosity, and no estimate of pore volume in given form the BTE SA measurements?

**Response**: An intra-particle diffusion model is not appropriate for OTC adsorption of kaolinite, so we have removed the contents about intra-particle diffusion model from the manuscript.

8. Page 10, I find the results and discussion of a diffusion based mechanism a little puzzling. Given that the porosity is low, and that kaolinite is a classic non-swelling clay, I find it extremely unlikely that there is a diffusion process at play. I do not think there is sufficient evidence to conclude that there is a movement of OTC form external to internal surfaces. Kinetic modeling of this type is really just a curve fitting process, and the models with more adjustable parameters will naturally provide a better fit. Conclusions about actual kinetic mechanisms really need more substantiation that just the fit of a particular semi-empirical model.

**Response**: According to some studies about kinetics of kaolinite adsorption, intra-particle diffusion process is very little. So it is not appropriate to use an intra-particle diffusion model here and the contents have been deleted from the manuscript.

9. Page 11, the commentary on the application of the isotherm modeling is similarly superficial as the kinetic modeling mentioned above, and hence I think there is an over-interpretation of the data sets based on the model parameters, though the overall all conclusion is rather weak in that the authors suggest that there are ‘multiple’ mechanisms responsible for the uptake.

**Response**: We have revised some detail description of isotherm model and discussion has been added.

10. Page 12, the IR spectra and the interpretation is very unconvincing. How were these spectra collected? Is this diffuse reflectance or ATR of dried samples or pastes? There is no description in the methods section, and it is simply not possible to make conclusions from the spectra unless more detail is known about what these spectra represent (ie wet/dry samples, ATR etc). What was the surface concentration of the OTC when absorbed, can you actually see it in the IR, was the OTC just crystalline sample or a solution etc?

**Response**: We have added the method of IR spectra collection in section 2.1. In this study, KBr pressed-disc method was adopted. OTC of 10mg/L adsorbed by kaolinite was used and all samples for IR measuring were solid.

References

1.	Yandan Li. Sorption behavior of typical flueroquinolone antibiotics on kaolinite：batch experiments. Bioresour Technol. Chia university of geosciences. 2017.
